# Simulation of a Human-Scale Cerebellar Network Model on the K Computer

**DOI:** 10.3389/fninf.2020.00016

**Published:** 2020-04-03

**Authors:** Hiroshi Yamaura, Jun Igarashi, Tadashi Yamazaki

**Affiliations:** ^1^Graduate School of Informatics and Engineering, The University of Electro-Communications, Tokyo, Japan; ^2^Head Office for Information Systems and Cybersecurity, RIKEN, Saitama, Japan

**Keywords:** cerebellum, human-scale model, computer simulation, spiking network model, K computer, MONET

## Abstract

Computer simulation of the human brain at an individual neuron resolution is an ultimate goal of computational neuroscience. The Japanese flagship supercomputer, K, provides unprecedented computational capability toward this goal. The cerebellum contains 80% of the neurons in the whole brain. Therefore, computer simulation of the human-scale cerebellum will be a challenge for modern supercomputers. In this study, we built a human-scale spiking network model of the cerebellum, composed of 68 billion spiking neurons, on the K computer. As a benchmark, we performed a computer simulation of a cerebellum-dependent eye movement task known as the optokinetic response. We succeeded in reproducing plausible neuronal activity patterns that are observed experimentally in animals. The model was built on dedicated neural network simulation software called MONET (Millefeuille-like Organization NEural neTwork), which calculates layered sheet types of neural networks with parallelization by tile partitioning. To examine the scalability of the MONET simulator, we repeatedly performed simulations while changing the number of compute nodes from 1,024 to 82,944 and measured the computational time. We observed a good weak-scaling property for our cerebellar network model. Using all 82,944 nodes, we succeeded in simulating a human-scale cerebellum for the first time, although the simulation was 578 times slower than the wall clock time. These results suggest that the K computer is already capable of creating a simulation of a human-scale cerebellar model with the aid of the MONET simulator.

## Introduction

Computer simulation of the whole human brain is an ambitious challenge in the field of computational neuroscience and high-performance computing (Izhikevich, 2005; Izhikevich and Edelman, 2008; Amunts et al., 2016). The human brain contains approximately 100 billion neurons. While the cerebral cortex occupies 82% of the brain mass, it contains only 19% (16 billion) of all neurons. The cerebellum, which occupies only 10% of the brain mass, contains 80% (69 billion) of all neurons (Herculano-Houzel, 2009). Thus, we could say that 80% of human-scale whole brain simulation will be accomplished when a human-scale cerebellum is built and simulated on a computer. The human cerebellum plays crucial roles not only in motor control and learning (Ito, 1984, 2000) but also in cognitive tasks (Ito, 2012; Buckner, 2013). In particular, the human cerebellum seems to be involved in human-specific tasks, such as bipedal locomotion, natural language processing, and use of tools (Lieberman, 2014). Once a human-scale cerebellar network model is built, the computer simulation of the model will be a useful tool to examine the roles of the cerebellum in such complex tasks.

Several attempts have been made to simulate large-scale spiking network models on supercomputers. A large-scale model of the cerebral cortex, with 1.51 billion neurons and 16.8 trillion synapses has been built on the Japanese flagship K computer (RIKEN Center for Computational Science) (Helias et al., 2012; Kunkel et al., 2014; Jordan et al., 2018). For the cerebellum, we previously built a cat-scale cerebellar model composed of 1 billion spiking neurons on another supercomputer (Yamazaki et al., 2019). Currently, in a national project called “Post-K exploratory challenge #4,” we have been building large-scale spiking network models of the cerebral cortex (Igarashi et al., 2019), basal ganglia (Moren et al., 2019), and cerebellum on the K computer.

In this study, we aimed to build and simulate a human-scale spiking network model of the cerebellum under the Post-K project, where we use the entire K computer system and our in-house spiking neural network simulator MONET (Millefeuille-like Organization NEural neTwork) (Igarashi et al., 2019). The MONET simulator is optimized to the K computer and its successor Fugaku (RIKEN Center for Computational Science). The simulator arranges a given large-scale spiking network on a two-dimensional layered sheet, and splits the sheet into a number of smaller tiles for parallel computing, where subnetworks on neighboring tiles can exchange spikes. This tile-based decomposition method achieves a good weak-scaling property, because it is enough for each tile to communicate with only the surrounding tiles. However, there is a strong assumption behind the tile-based decomposition: the target network must consist of mostly short-range connections and few long-range connections. In other words, a target brain region must suffice for a characteristic anatomical structure to be simulated efficiently on the MONET simulator.

The cerebellum is known for its regular and repeated crystallized anatomical structure (Eccles et al., 1967), which seems to be ideal for tile-based decomposition. Therefore, we built our previous spiking network model of the cerebellum on the MONET simulator while extending the network size. Owing to the characteristic structure of the cerebellum, we achieved good weak-scaling from 1,024 nodes to 82,944 nodes on the K computer. Our model is not just a baseline model showing only spontaneous activity, but a functional model that can reproduce a basic eye movement reflex task called optokinetic response (OKR). Eventually, using all 82,944 nodes of the K computer, we succeeded in simulating a human-scale cerebellar model with 68 billion neurons.

## MATERIALS AND METHODS

### K Computer

Until its shutdown on August 30th, 2019, the K computer (RIKEN Center for Computational Science) was the Japanese Flagship supercomputer. We used this computer for our cerebellar simulation. An overview of the K computer is described elsewhere (Miyazaki et al., 2012). Briefly, the K computer comprised 82,944 compute nodes and 1.26 petabyte of DRAM memory in total. The peak performance was 11.3 PFLOPS. Each node had a SPARC VIIIfx CPU with 8-cores operating at 2 GHz and 16 GB of DRAM memory. The compute nodes were interconnected via six-dimensional torus called “Tofu” network. OpenMP v3.0 and MPI v2.1 were supported for parallel programming.

### Neural Network Simulator MONET

**FIGURE 1:**
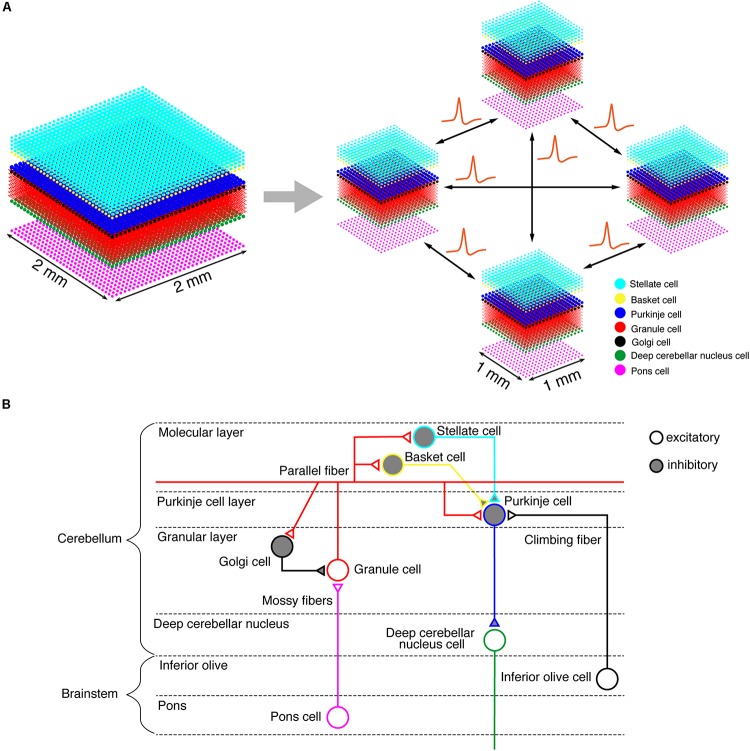
Building a cerebellar neural network model. **(A)** Schematics of the tile structure composed of two-dimensional sheets of neural networks. We show the three-dimensional structure of our cerebellar network model. Dots indicate neurons. A 2 mm×2 mm of the cerebellar neuronal sheet on a tile (left) is partitioned into regular square tiles (right). Each tile communicates with the neighboring tiles to exchange spike data. **(B)** A schematic of the cerebellar cytoarchitecture. The cerebellum receives two types of afferents from pons cells and inferior olive cells, respectively.

On the K computer, we ran a versatile spiking neuron network simulator called MONET (Millefeuille-like Organization NEural neTwork) (Igarashi et al., 2019). The MONET simulator is designed to run on multi-node clusters such as the K computer. The simulator computes dynamics of a given large-scale spiking network model in parallel by partitioning the entire network into a number of small subnetworks called “tiles,” assigning a tile to a processor, and performing numerical calculation on each processor independently while exchanging spikes across processors (Figure 1A). A tile can stack an arbitrary number of layered sheets in *z*-axis, which is suitable for building layered sheet types of neural networks naturally, such as the cerebellar cortex with three layers (granular layer, Purkinje cell layer, and molecular layer).

A typical use case of the simulator is as follows. First, users prepare parameter files in a JSON format that defines the simulation settings and neural network parameters, respectively. The former includes simulation time, the number of nodes to use, the size of a tile, and the output file name (Supplementary Figure S1). The latter describes layer thickness, the number of neurons in a tile, types of neurons, and connectivity among neurons (Supplementary Figure S2). The neuron parameters include membrane time constant, threshold, resting and reset values of membrane potential, external current (I_ex), and absolute refractory period. The connectivity parameters specify projection area, connection probability, receptor time constant, receptor reversal potential, and synaptic weight. Second, a python script generates intermediate files for all compute nodes from the JSON file. These intermediate files are used to build a network on the MONET simulator. The core of the MONET simulator is a C program that reads the files, executes the simulation using a forward Euler method with fixed time step of 0.1 ms, and generates output data. These data include computational time, spike time and its neuron ID, and neuron position in three-dimensional space.

### Neural Network Model of the Cerebellum

We implemented a spiking network model of the cerebellum that we had developed previously (Figure 1B; Yamazaki and Nagao, 2012) on the MONET simulator. The model receives two afferent inputs from mossy fibers and climbing fibers. We implemented one square millimeter of the cerebellar neuronal sheet on a tile, and the number of tiles was increased to extend the network size in the *x*–*y* plane and the depth along the *z*-axis. Along the *z*-axis, we stacked seven layers: upper and lower molecular layers, Purkinje cell layer, granular layer, deep cerebellar nucleus, inferior olive, and pons with thicknesses of 0.1, 0.1, 0.03, 0.2, 0.1, 0.1, and 0.2 mm, respectively (Eccles et al., 1967). A layer can have multiple stacked sheets along the *z*-axis. Figure 1A shows the three-dimensional structure of our cerebellar network model. The upper molecular layer was composed of four sheets of stellate cells (STs), and each sheet contained 32 × 32 STs. The deep molecular layer and the Purkinje cell layer were a single sheet containing 32 × 32 basket cells (BAs), and Purkinje cells (PCs), respectively. The granular layer was composed of eight sheets of granule cells (GRs) and a sheet of Golgi cells (GOs). A GR sheet contained 320 × 320 GRs, and a GO-sheet 32 × 32 GOs. The deep cerebellar nucleus and the pons were a single sheet with 32 × 32 deep cerebellar nucleus cells (DCNs) and pons cells, respectively. The inferior olive layer contains only one sheet with one inferior olive cell (IO). Table 1 summarizes the total numbers of neurons for a tile. These numbers were set based on previous experimental data (Lange, 1974, 1975; Ito, 1984; Harvey and Napper, 1991; Heckroth, 1994). Neurons were modeled as conductance-based leaky integrate-and-fire units. Parameters were set based on our previous studies. Table 2 summarizes each neuron’s parameters.

**TABLE 1:** Numbers of neurons per tile (1mm^2^) in each cerebellar layer.

	**Numbers**	**Experimental data (neuron density)**
ST	4,096	18,695 neurons/mm^3^ (Ito, 1984)
BA	1,024	6,577 neurons/mm^3^ (Ito, 1984)
PC	1,024	330-650 neurons/mm^2^ (Lange, 1975; Ito, 1984; Harvey and Napper, 1991)
GR	819,200	1.75-2.8×10^6^ neurons/mm^3^ (Lange, 1975; Ito, 1984)
GO	1,024	629-740 neurons/mm^3^ (Lange, 1974)
DCN	1,024	21,078 neurons/mm^3^ (Heckroth, 1994)
IO	1	
Pons	1,024	

*Lange (1974, 1975), Ito (1984), and Harvey and Napper (1991) report anatomical data in various species. In the table, we refer to the cat data of the references, because the present model was based on the cat data, as in previous models (Yamazaki and Nagao, 2012; Yamazaki et al., 2019). Heckroth (1994) reports data in the mouse. ST, stellate cell; BA, basket cell; PC, Purkinje cell; GR, granule cell; GO, Golgi cell; DCN, deep cerebellar nucleus cell; IO, inferior olive cell.*

**TABLE 2:** Neuron parameters.

	**ST**	**BA**	**PC**	**GR**	**GO**	**DCN**	**IO**	**Pons**
Membrane time constant (ms)	10	10	10	7.2	12	10	10	10
Threshold (mV)	−55	−55	−50	−35	−50	−40	−50	−50
Reset value (mV)	−70	−70	−70	−70	−70	−70	−70	−70
Resting membrane potential (mV)	−70	−70	−70	−58	−70	−70	−70	−70
I_ex (nA)	0	0	22	0	0	32	50	24
Absolute refractory period (ms)	1	1	1	1	1	1	1,500	1

*ST, stellate cell; BA, basket cell; PC, Purkinje cell; GR, granule cell; GO, Golgi cell; DCN, deep cerebellar nucleus cell; IO, inferior olive cell.*

Anatomical connections among neurons were made according to the known anatomical structure (Eccles et al., 1967; Apps and Garwicz, 2005; Barmack and Yakhnitsa, 2008a). In the MONET simulator, connections were set as a two-dimensional Gaussian with parameters of the projection area and connection probability. Table 3 summarizes setting of connection probability at peak and sigma of a two-dimensional Gaussian function. Notably, implementing connections via parallel fibers needs special care, because parallel fibers do not extend radially but rectangularly. The parallel fiber connections were set by providing the width (pre_width), the length (post_width), and connection probability as in Supplementary Figure S3. Table 4 summarizes the parameters. Neurons have synapses with α-amino-3-hydroxy-5-methyl-4-isoxazolepropionic acid (AMPA) and N-methyl-D-aspartate (NMDA) or γ-aminobutyric acid type A (GABAA) and γ-aminobutyric acid type B (GABAB). Synapse dynamics were described by alpha functions. The time constants are summarized in Table 5. Each synaptic connection has its connection weight. The weight values are summarized in Table 6.

**TABLE 3:** Intra-regional connection for two-dimensional Gaussian function.

**Pre**	**GO**	**IO**	**Pons**
**Post**	**GR**	**PC**	**GR**
Probability at peak	0.04	1	1
Sigma (μm)	200	350	25
References	Crowley et al., 2009; Hull and Regehr, 2012	Eccles et al., 1967; Ito, 1984	Eccles et al., 1967; Ito, 1984

*ST, stellate cell; BA, basket cell; PC, Purkinje cell; GR, granule cell; GO, Golgi cell; DCN, deep cerebellar nucleus cell; IO, inferior olive cell.*

**TABLE 4:** Intra-regional connection for orthogornal_cross function.

**Pre**	**ST**		**BA**		**PC**		**GR**			
**Post**	**ST**	**PC**	**BA**	**PC**	**BA**	**DCN**	**ST**	**BA**	**PC**	**GO**
**Value to set in MONET**										
Pre_width (μm)	50	50	50	50	50	75	500	500	500	250
Post_width (μm)	200	200	200	200	200	600	100	100	100	100
**Projection area (pre cell)**										
Mediolateral (μm)	100	100	100	100	100	150	1000	1000	1000	500
Rostrocaudal (μm)	200	200	200	200	200	600	100	100	100	100
Probability	0.02	0.1	0.02	0.1	0.05	0.3	0.05	0.05	0.05	0.025
References	Kim et al., 2014; Rieubland et al., 2014	Dizon and Khodakhah, 2011	Kim et al., 2014; Rieubland et al., 2014	Dizon and Khodakhah, 2011	Eccles et al., 1967; Ito, 1984	Person and Raman, 2012	Isope and Barbour, 2002; Barmack and Yakhnitsa, 2008b	Isope and Barbour, 2002; Barmack and Yakhnitsa, 2008b	Isope and Barbour, 2002; Barmack and Yakhnitsa, 2008b; Walter et al., 2009	Eccles et al., 1967; Ito, 1984

*ST, stellate cell; BA, basket cell; PC, Purkinje cell; GR, granule cell; GO, Golgi cell; DCN, deep cerebellar nucleus cell; IO, inferior olive cell.*

**TABLE 5:** Time constants for synapses.

**Synapse**	**Time constant (ms)**
AMPA	2
NMDA	100
GABA_A_	2
GABA_A_ (GO to GR)	10

*The parameters were set based on our previous studies (Yamazaki and Nagao, 2012; Yamazaki et al., 2019). AMPA, α-amino-3-hydroxy-5-methyl-4-isoxazolepropionic acid; NMDA, N-methyl-D-aspartate; GABA_*A*_, γ-aminobutyric acid type A; GO, Golgi cell; GR, granule cell.*

**TABLE 6:** Synaptic weights.

	**Postsynaptic neuron**							
	**ST**	**BA**	**PC**	**GR**	**GO**	**DCN**	**IO**	**Pons**
**Presynaptic neuron**								
ST	0.02		0.05					
BA		0.02	0.1					
PC		0.01				0.0025		
GR	0.00145	0.00145	0.0013		AMPA			
					0.0008			
					NMDA			
					0.00017			
GO				3.0				
DCN								
IO			0.1					
Pons				0.5				

*ST, stellate cell; BA, basket cell; PC, Purkinje cell; GR, granule cell; GO, Golgi cell; DCN, deep cerebellar nucleus cell; IO, inferior olive cell.*

### Numerical Simulation of the Cerebellar Model

First, we examined the resting state activity of the model in response to spontaneous mossy fiber input signals of 8 Hz. We measured the scalability of the simulation described below. Then, as a benchmark test to confirm the network dynamics consistent with experimental data, we performed a computer simulation of a simple cerebellum-dependent eye movement control task called OKR. The OKR is an eye movement reflex, which is induced by slow movement of the whole visual field image on the retina. The cerebellum issues the motor command for eyes to move to the same direction with the visual field movement, so that the blur in the retinal image is reduced (Shutoh et al., 2006). In the OKR, the cerebellum receives the information of the moving speed of the visual world from nucleus reticularis tegmenti pontis (NRTP), and issues the motor command from vestibular nucleus (VN) cells. In our model, NRTP was composed of 32 × 32 cells aligned on two-dimensional grids. For NRTP cells, we set the membrane time constant, threshold, reset value, resting membrane potential, and absolute refractory period to 40 ms, −60 mV, −70 mV, −70 mV, and 1 ms, respectively. A two-dimensional Gaussian function was used for connection from NRTP cells to GRs. The connection probability at peak and sigma of the two-dimensional Gaussian function were set to 0.1 and 75, respectively. On the other hand, PCs project to VN cells. The VN was composed of 32 × 32 cells aligned on two-dimensional grids. We fed sinusoidally modulating I_ex into NRTP cells. I_ex of PCs and VN cells were set to 24 and 40 nA, respectively. We set synapse weights as shown in Supplementary Table S1. The other parameters were the same as the cerebellar network model described above.

### Analysis of Spike Patterns of Granule Cells

A characteristic feature of our cerebellar model is spatiotemporal combinatorial encoding of mossy fiber input signals by population activity of a number of granule cells via random inhibitory connections with Golgi cells in the granular layer (Yamazaki and Tanaka, 2005, 2007a,b; Yamazaki and Nagao, 2012). In response to sustained mossy fiber signals, granule cells reveal different temporal activity patterns. The cells undergo random repetition of transitions between burst and silent states. The burst state is sustained for tens to hundreds of milliseconds. Because different granule cells show different temporal activity patterns, the population of active granule cells changes gradually in time (Supplementary Figure S4A). To quantify the gradual change of the active granule cell population, we employ a measure called “similarity index” defined previously.

We assume that the simulation time is discretized with temporal resolution *dt* (=0.1 ms). Let *f*_*i*_(*t*) be the spike activity of model GR *i* at time *t* (ms), thus *f*_*i*_(*t*) = 1 (GR *i* elicits a spike at time *t*), *f*_*i*_(*t*) = 0 (otherwise). Then, let *z*_*i*_(*t*) be the temporal trace of the activity of GR *i* (i.e., EPSPs on a PC) decayed exponentially with time constant τ_*PC*_ as follows (Yamazaki and Tanaka, 2007b):

(1) $$z_{i}\,\left(t\right)=\frac{1}{\tau_{P\,C}}\,\sum_{s=0}^{t}e\,x\,p\,\left(-\frac{t-s}{\tau_{P\,C}}\right)\,f_{i}\,\left(s\right)$$

where τ_*PC*_ was set at 50 ms. Here, the sum with respect to *s* is a discretized version of temporal integration over *s* with *dt*. We defined the similarity index *SI* (Δ*t*) as follows (Yamazaki and Tanaka, 2007b):

(2) $$S\,I\,\left(\text{Δ}\,t\right)=\frac{1}{T}\,\sum_{t=0}^{T}\frac{\sum_{i}z_{i}\,\left(t\right)\,z_{i}\,\left(t+\text{Δ}\,t\right)}{\sqrt{\sum_{i}z_{i}^{2}\,\left(t\right)}\,\sqrt{\sum_{i}z_{i}^{2}\,\left(t+\text{Δ}\,t\right)}}$$

where *T* represents the temporal duration to calculate the similarity. Here again, the sum with respect to *t* denotes a discretized version of temporal integration over *t* with Δ*t*. We set *T* at 2 s. The similarity index basically calculates the normalized autocorrelation for two GR populations at time *t* and *t* + Δ*t* over *t*. The numerator calculates the correlation, whereas the denominator normalizes the value between 0 and 1. In other words, this index represents how the two GR populations separated by Δ*t* are similar on average. If the value is 1, the populations are identical, whereas if it is 0, they are completely different. Section Supplementary Material S1 provides a more detailed explanation.

### Measuring Scaling Property

Weak scaling and strong scaling are two important measures to quantify the performance of numerical simulation. In weak scaling, we increase the size of the neural network model (i.e., the numbers of neurons and synapses) while increasing the number of compute nodes involved, where the computational load per node is kept the same. Perfect weak scaling yields that the computational time remains unchanged across any network size. In other words, weak scaling ensures that we can simulate arbitrarily large-scale network models as long as sufficient computational power is provided. This way, human-scale or even larger simulations are possible. On the other hand, in strong scaling, the number of compute nodes is varied while fixing the network size. Perfect strong scaling yields that the computational time halves by doubling the computational power. In other words, strong scaling ensures that the computer simulation can be faster as more computational power is adopted.

To accomplish human-scale simulation, the weak scaling property is important. Therefore, we examined the weak scaling property of our simulation.

## Results

### Network Dynamics

In the cerebellar network model, GRs in the granular layer receive external input from Pons cells (Figure 1B). First, we examined the network dynamics in response to spontaneous discharge in the Pons, which were firing at 8 Hz. In the spontaneous discharge condition, the average firing rate of STs, BAs, PCs, GRs, GOs, and DCNs were 14, 14, 55, 1.9, 0.10, and 27Hz, respectively. In Table 7, we compared the simulated firing rates and the experimental data in rodents.

**TABLE 7:** Firing rates during resting state.

	**Simulated firing rates**	**Experimental data in rodents**
ST	14Hz	14.9Hz (Blot et al., 2016), 20Hz (Jelitai et al., 2016)
BA	14Hz	14.9Hz (Blot et al., 2016), 20Hz (Jelitai et al., 2016)
PC	55Hz	48.9Hz (Blot et al., 2016), 66.9Hz (Jelitai et al., 2016)
GR	1.9Hz	0.12Hz (Powell et al., 2015), 0.14Hz (Jelitai et al., 2016), 1.9Hz (Chen et al., 2017)
GO	0.10Hz	2–25Hz (Holtzman et al., 2006), 7.53Hz (Dugué et al., 2009)
DCN	27Hz	10Hz (Menardy et al., 2019), 36.6Hz (LeDoux et al., 1998)

*The experimental data indicate the average firing rates reported in the reference, except for Holtzman et al. (2006). For Holtzman et al. (2006), it is shown as the firing frequencies in the range. ST, stellate cell; BA, basket cell; PC, Purkinje cell; GR, granule cell; GO, Golgi cell; DCN, deep cerebellar nucleus cell.*

**FIGURE 2:**
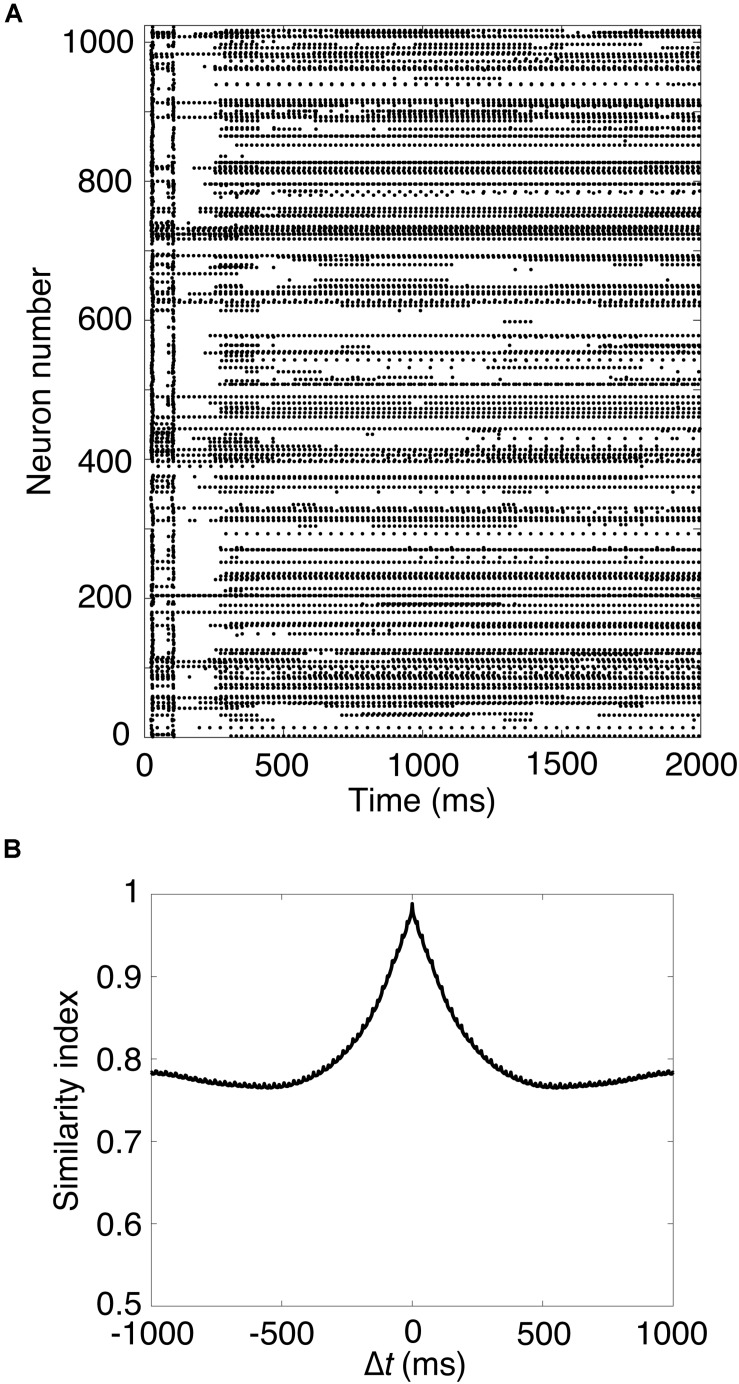
Spike patterns of granule cells **(A)** Activity pattern of 1,024 granule cells chosen randomly in response to constant input signals for 2,000 ms. Horizontal axis is time (ms) and vertical axis is neuron number. Each dot represents a spike. **(B)** Similarity index for the spike patterns of granule cells.

Next, we carried out a simulation in Pons cells issuing spikes in a high frequency, which were firing at 50 Hz. Figure 2A shows a raster plot of GRs in response to constant strong input signals in the Pons. GRs repeatedly emitted spikes in bursts at random time intervals, then stopped suddenly. Different GRs showed different temporal activity patterns. To confirm the property, we calculated the similarity index by Eq. 2 and plotted it in Figure 2B. The similarity index monotonically decreases as Δ*t* increases. The result suggests that the population of active GRs changes gradually and slowly with time, and any population would appear only once during a cycle of the input signals (see Supplementary Material S1). This activity pattern emerged from the random recurrent inhibitory network, which was composed of GRs and GOs. These data reproduced the reservoir-like activity pattern of GRs demonstrated in previous studies (Yamazaki and Tanaka, 2007a).

**FIGURE 3:**
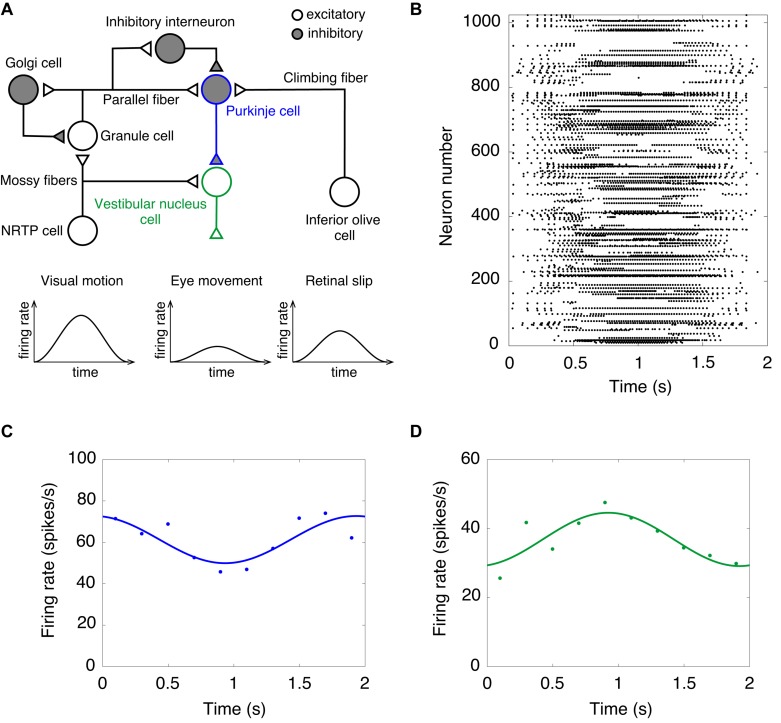
A computer simulation of the optokinetic response (OKR). **(A)** Schematics of the cerebellar neural circuitry for OKR. Note that input from NRTP cells to vestibular nucleus cells and retinal slip are ignored in this simulation of the OKR. NRTP, nucleus reticularis tegmenti pontis. **(B)** Activity pattern of 1,024 granule cells chosen randomly in response to input signals of OKR for 2 s. Horizontal axis is time (s) and vertical axis is neuron number. Dot represents a spike. **(C)** Firing rate of Purkinje cells. **(D)** Firing rate of vestibular nucleus cells. In **(C,D)**, data of firing rate show the mean of 1,024 cells (dots). Lines show fitting the data with cosine functions. Horizontal axis is time (s) and vertical axis is firing rate (spikes/s).

Finally, we performed a computer simulation of the OKR. In the OKR, the cerebellar cortex receives two input signals (Figure 3A). Visual motion information is transmitted from the retina to the GRs through mossy fibers via NRTP. Information of retinal slip is transmitted to PCs through climbing fibers via the IO. The activity of VN cells that are the only output cells of the cerebellum correspond to eye movement (Figure 3A). We fed sinusoidally modulating external current like the visual motion pattern of Figure 3A into NRTP cells. NRTP cells exhibited sinusoidally modulating firing rates similar to a visual motion pattern (data not shown). Figure 3B shows the raster plot of GRs in response to the input signals in NRTP cells. We found that GRs exhibited a reservoir-like activity pattern. Figures 3C,D show the firing rate of PCs and VN cells, respectively. PCs modulated the firing rate out of phase with the firing rate of NRTP cells. The result corresponds to Figure 3A of a previous study by Yamazaki and Nagao (2012). In that figure, the modulation range of the firing frequency in PCs at the 100th cycle was 50–80 Hz. Our current simulation had the same modulation range as seen in previous study (Yamazaki and Nagao, 2012). The neural activity of PCs during the OKR has been recorded in various animals. The following shows the modulation ranges of the firing frequency in PCs in each study; 30–50 Hz (rabbit, Figure 3C in Nagao, 1988), 40–100 Hz (monkey, Figure 3C in Sato and Noda, 1992), 20–100 Hz (cat, Figure 2A in Kitama et al., 1999), 40–100 Hz (mouse, Figure 3A in Yoshida et al., 2007). The simulation result in this study is within the range of the results in the animal studies. Furthermore, VN cells modulated the firing rate out of phase with the firing rate of PC cells due to disinhibition. This result corresponds to Figure 3B in a previous study by Yamazaki and Nagao (2012). In that figure, the modulation range of the firing frequency in VN cells at the 100th cycle was 30–90 Hz. In this simulation, the modulation range of the firing frequency in VNs was 20–50 Hz. Because the MONET simulator does not implement the plasticity rule, we did not investigate OKR gain adaptation. This issue will be discussed as a limitation in section “Discussion.” Because activity of IO cells plays an important role in adaptation and is also too low, we did not feed sinusoidally modulating external current into the IO; therefore, we ignored inputs from retinal slip in this study.

### Weak Scaling Property

**FIGURE 4:**
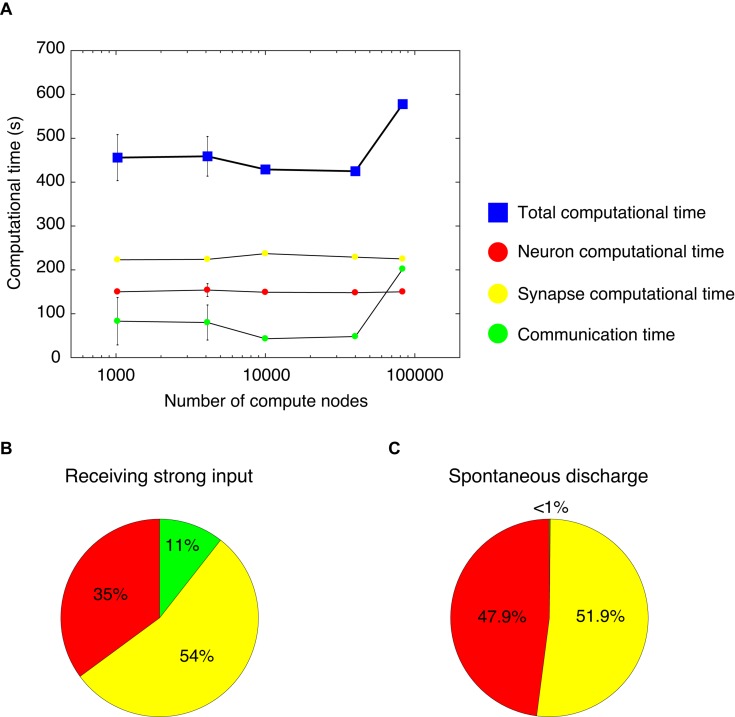
Computational time in the cerebellar network model. **(A)** Weak scaling property. We varied the number of compute nodes: 1,024, 4,096, 10,000, 40,000, and 82,944. The horizontal axis is the number of compute nodes, and the vertical axis is the computational time (s) spent for 1 s simulation. The total computational time (blue square) = calculation time of membrane potentials (neuron computational time, red circle), calculation time of synaptic inputs (synapse computational time, yellow circle), and calculation time of communication between nodes (communication time, green circle). With 1,024 and 4,096 nodes, we carried out five simulations. For each node, we obtained the total computational time of 456±52.4, 459±45.2, 429, 425, and 578 s, respectively (blue squares). The neuron computational times were 150±3.03, 154±14.6, 149, 148, and 150 s, respectively. The synapse computational times were 223±3.01, 224±2.23, 237, 229, and 225 s, respectively. The communication times were 83±54, 80±40, 43, 48, and 202 s, respectively. The data for 1,024 and 4,096 nodes are displayed as mean ± standard deviation. The horizontal axis is shown in log-scale. **(B)** A pie chart of the components of computational time with 1,024 nodes. **(C)** A pie chart of the components of computational time with 1,024 nodes for the cerebellar network model with spontaneous discharge.

We measured computational time while varying the number of compute nodes from 1,024–4,096 to 10,000–40,000 and finally 82,944, and while increasing the network size of the cerebellar network model. Thus, we analyzed the weak-scaling property of the cerebellar network model (Figure 4A). With 1,024 and 4,096 nodes, we changed the seed of the random number and carried out five simulations. Due to limited resources, it was not possible to carry out several simulations on more than 10,000 nodes. For each node, we obtained a computational time of 456, 459, 429, 425, and 578 s, respectively (blue squares, Figure 4A). The computational time did not increase until 40,000 nodes. However, at 82,944 nodes, the computational time increased by 1.5×. Nevertheless, this represents a good scaling property in this model.

The computational time was calculated for membrane potentials (neuron computational time), calculation of synaptic inputs (synapse computational time), and communication via network to exchange spike information among different computational nodes (communication time). Figure 4A shows each computational time at each node. From 1,024 to 82,944 nodes, there were no increases in neuron and synapse computational times; however, there was an increase in communication time at 82,944 nodes. The increase in the total computational time at 82,944 nodes was due to the increase in communication time. Note that an increase in communication time was observed under some setting conditions at 1,024 and 4,096 nodes. In the MONET simulator, the cerebellar two-dimensional sheet is partitioned into regular square tiles and assigned to compute nodes on the K computer. There is a possibility that the tiles that require communication were distantly assigned to compute nodes. Therefore, the assignment may influence the communication time. The issue related to optimally assigning tiles to compute nodes will be addressed in section “Discussion.”

Using the full nodes (82,944 nodes) of the K computer, the total number of neurons in the human-scale cerebellar network model was 68,627,284,992 (approximately 68 billion), which is comparable to the human cerebellum (Herculano-Houzel, 2009). Furthermore, in the human-scale cerebellar network model, the total number of synapses was 5,389,950,000,000 (approximately 5.4 trillion). Finally, we examined the occupying memory for the human-scale cerebellar network model. In the K computer, each node has 16 GB of DRAM memory. In the simulation of the human-scale cerebellar network model, up to 9.6 GB of memory per node was occupied, then the total memory was 0.80 PB (9.6 GB per node × 82,944 nodes).

We further examined the breakdown of computational time. Figure 4B shows a pie chart of the different components of the computational time with 1,024 nodes, which took 416 s to simulate 1 s of biological time. The neuron, synapse, and communication times were 35, 54, and 11%, respectively. The cerebellar network model for Figures 4A,B received constant strong inputs, indicating high activity in the whole network. Next, we sought to investigate the computational time when the activity in the whole network was low. We hypothesized that a lower activity state in the network model corresponded to spontaneous discharge. Therefore, we created a cerebellar network model with spontaneous discharge and performed the simulation with 1,024 nodes. The cerebellar network model with spontaneous discharge took 403 s to simulate 1 s of biological time, which was decreased compared with the cerebellar network model that received constant strong inputs (416 s). Figure 4C shows a pie chart of the different ratios of computational time per component. The neuron, synapse, and communication computational times were 47.9, 51.9, and <1%, respectively. The communication time in the cerebellar network model with spontaneous discharge was negligible compared with the cerebellar network model receiving constant strong inputs. This was due to fewer emitted spikes in the cerebellar network model with spontaneous discharge; therefore, communication among nodes was reduced substantially. These results suggest that the MONET simulator implements efficient communication mechanisms.

## Discussion

### Computer Simulation of a Human-Scale Cerebellar Network Model

In this study, we built a cerebellar network model based on electrophysiological and anatomical data and carried out the simulation on the K computer using the MONET simulator. We carried out computer simulation of resting-state activity driven by spontaneous mossy fiber input signals as well as network dynamics during OKR. In the latter, we reproduced a reservoir-like activity pattern of granule cells and observed similar activity patterns in animal experiments (Yamazaki and Nagao, 2012). We also confirmed a good weak-scaling property from 1,024 to 82,944 computational nodes on the K computer. Using the full nodes (82,944 nodes) of the K computer, we were able to simulate a human-scale cerebellar model composed of approximately 68 billion neurons.

What can be done with such human-scale cerebellar simulation? Various potential usages are considered. Regarding its functional roles, the cerebellum is divided into functional modules called microcomplexes (Ito, 1984; Apps and Garwicz, 2005; Apps et al., 2018). A recent study using fMRI has demonstrated that the human cerebellum has functional parcellations in various tasks (King et al., 2019). It is assumed that even simple reflexive motor task such as OKR involve multiple microcomplexes of neural activity. Furthermore, a recent study in mice reports that performance of complex cognitive tasks involves widespread regions of the cerebral cortex (Pinto et al., 2019). Because the cerebellum interacts with those cortical regions (Buckner et al., 2011; Guell et al., 2018) in complex tasks in humans, it is assumed that widespread regions of the cerebellum engage with widespread cortical regions. Therefore, to explore how multiple regions in the human cerebellum interact to accomplish tasks, large-scale cerebellum simulation and even human-scale whole-brain simulation will be useful. Meanwhile, the connectome data from the human brain have been made available gradually. To incorporate human connectome data, a human-scale network model will be necessary. Once we succeed in adopting the human-level connectome data, we would reproduce the cerebellar activity during human-specific cognitive tasks. Eventually, a human-scale cerebellar model will be part of the human-scale whole brain simulation, which will be discuss later.

### Current Limitations and Future Extensions

Although the present model is built based on known electrophysiological and anatomical data (Yamazaki and Nagao, 2012) and the size is unprecedented, several important features are missing. In the cerebellum, the plasticity at parallel fiber-Purkinje cell synapses plays prominent roles (Marr, 1969; Albus, 1971; Ito, 2001; Ito et al., 2014; Yamazaki et al., 2015; Yamazaki and Lennon, 2019). Moreover, there are various forms of synaptic plasticity distributed within the cerebellum (D’Angelo, 2014). Related to this plasticity, detailed synapse dynamics involving receptors and transporters such as metabotropic glutamate receptors and Ca^2+^ channels are also missing. Because these receptors and transporters contribute to the signaling processes in dendrites (Tank et al., 1988; Eilers et al., 1995; Ito, 2001, 2002), multi-compartment neuron models rather than single-compartment models used in this study must be taken into account. These are the future extensions that we aim to address.

Another limitation of the present simulation is the computational time. Even the simple simulation of resting state activity took 578 times more time than the wall clock time. This means that a computer simulation of cerebellar activity for just 1 min takes almost 10 h to complete. Such a slow computational time significantly constrains our research. For example, cerebellum-dependent motor learning takes at least a few hours or even days (Shutoh et al., 2006). Computer simulation of such learning is practically impossible. To accelerate computer simulation, special-purpose hardware for parallel computing such as graphics processing units (GPUs) provide tremendous computational power for cerebellum simulation. Yamazaki and Igarashi (2013) built a spiking network model of the cerebellum with 0.1 million neurons on GPU, and succeeded in simulating 1 s of biological time within 1 s of wall clock time (real-time simulation). Later, Yamazaki et al. (2019) employed the supercomputer Shoubu, which was composed of 1,024 PEZY-SC processors, and built a cat-scale cerebellar model with one billion spiking neurons enabling real-time simulation. Some cerebellar models are demonstrated in adaptive robot control (Garrido et al., 2013; Casellato et al., 2015). The next-generation Japanese flagship supercomputer, Fugaku, will have 100 times more computational capability than the K computer. The MONET simulator on Fugaku will be able to accomplish real-time simulation. Pursuing real-time simulation is another future extension.

Scaling property is also an issue. The MONET simulator successfully simulated the cerebellar network model with perfect weak scaling property until 40,000 nodes. However, the computational time increased with 82,944 nodes. This increase was due to a large increase in communication time. There are some possible causes for this increase in communication time. First, mapping the cerebellar two-dimensional sheet on the three-dimensional space of compute nodes interconnected by Tofu in the K computer influences communication time. There is a possibility that continuity between neighboring tiles is not preserved in the placement of the tiles on nodes. Therefore, distantly placed tiles may increase the communication time because of unnecessary distant communication and communication conflicts, especially when using large numbers of nodes. This may be relieved by mapping the sheets on the compute nodes while preserving their continuity. Second, there may be an increase in node synchronization latency. Subtle load unbalancing between nodes due to fluctuating neural activity may occur with small numbers of nodes; however, its effect on total elapsed times will increase as the number of nodes increases because synchronization requires a longer time for larger numbers of nodes. This problem might be solved in a supercomputer with a different topological network, such as a fat tree. Optimizing the simulator for Fugaku will be another important extension.

### Comparison With Other Large-Scale Models

Modern supercomputers allow cerebellar researchers to build large-scale network models. The present model is at one end of the spectrum: the network size is large, but the neuron model is simple (i.e., point neurons). At the other end, the neuron model is elaborated (i.e., multi-compartment neurons), but the network size is not so large. Several researchers have built models with the latter makeup. Solinas et al. (2010) reported that center-surround receptive fields for granule cells as a spatiotemporal filter emerged from the network dynamics with Golgi cells. Billings et al. (2014) investigated the capability of lossless sparse encoding within the granular layer. Cayco-Gajic et al. (2017) examined information capacity of parallel fiber inputs on a Purkinje cell. Sudhakar et al. (2017) demonstrated synchronized oscillation between granule cells and Golgi cells in the granular layer. Multi-compartment neuron models make the network model more realistic, whereas due to the heavy computational load, the network size cannot be large. In fact, multi-compartment neuron models are used to study detailed intracellular dynamics of a single neuron (De Schutter and Bower, 1994a, b; Roth and Häusser, 2001; Steuber et al., 2007; Zang et al., 2018). Thus, there is a trade-off between the network size and the details of single neurons. To address this problem, Casali et al. (2019) proposed a scaffold cerebellar model, which aims to compromise the network size and neuron models.

On the cerebral cortical simulation, Izhikevich and Edelman reported a simulation of a thalamocortical circuit that has hundred billion neurons and almost one quadrillion synapses (Izhikevich, 2005; Izhikevich and Edelman, 2008), but the details were not described. Using the K computer, Kunkel et al. (2014) and Jordan et al. (2018) built a balanced random cortical network model composed of 80% excitatory and 20% inhibitory integrate-and-fire neurons using the NEST simulator (Gewaltig and Diesmann, 2007). These studies used the full nodes of the K computer, and in Jordan et al. (2018), the model was composed of 1.51 billion neurons and 16.8 trillion synapses, which is one tenth of the number of neurons in the human cortex (Herculano-Houzel, 2009). However, the model took approximately 800 s to simulate 1 s of the biological time. Using the MONET simulator, Igarashi et al. (2019) built a cortex model with 6.04 billion neurons and 24.5 trillion synapses, which is one third of the human cortex, on 63,504 nodes of the K computer, and the simulation took 322 s for simulation of 1 s biological time. Because the MONET simulator exhibits a good weak-scaling property (Igarashi et al., 2019), the simulator would simulate the cortex model of the same size with Jordan et al. (2018) twice times faster.

Besides these previous studies, ours is the first report of a cerebellar network model showing realistic behaviors with a good weak scaling property up to the human scale. In particular, the weak scaling property was realized by the crystallized anatomical structure of the cerebellum, which seems optimal for the tile-based decomposition method adopted in the MONET simulator.

### Toward Building a Whole-Brain Network Model

The cerebellum is interconnected with other brain regions; the cerebral cortex and cerebellum form the cerebro-cerebellar communication loop (Kelly and Strick, 2003; Bostan et al., 2013; Proville et al., 2014; Voogd, 2014). In this loop, cerebellar outputs reach the primary motor and premotor cortex through the thalamus. Further, the cerebellum receives information from the primary motor and premotor cortex through the pons. Recent studies have demonstrated that the cerebellar-thalamo-cortical system plays an important role in motor control (Viaro et al., 2017; Nashef et al., 2018, 2019) and motor learning (Tanaka et al., 2018). A recent study also demonstrated that the cortico-cerebellar loop contributes to cognitive processes (Gao et al., 2018). In immobile animals, synchronization between cerebellar local field potentials (LFPs) and sensorimotor cortical LFPs within the theta frequency range has been shown (Courtemanche and Lamarre, 2005). To understand brain function, we need to consider interaction with multiple brain regions. We have developed a spiking neural network model of the primary motor cortex (Igarashi et al., 2019) and thalamus (unpublished data) using the MONET simulator. This simulator can connect the cerebellar network and thalamo-cortical network models (unpublished data). Our cerebellar-thalamo-cortical network model might advance understanding of the functional interaction of the cerebral cortex and cerebellum, and the mechanism of synchronization. In addition, previous studies have demonstrated that the cerebellum and basal ganglia are interconnected (Hoshi et al., 2005; Bostan et al., 2010, 2013; Bostan and Strick, 2018). Recent studies have also demonstrated that the cerebellar output influences the neuronal activity of the basal ganglia and basal ganglia-dependent behavior (Chen et al., 2014; Xiao et al., 2018). To examine roles of these inter-regional communications in both motor and cognitive functions, a whole-brain model will be necessary.

Furthermore, the brain has multiple learning systems. Particularly, the cerebral cortex, cerebellum, and basal ganglia are considered unsupervised, supervised, and reinforcement learning system, respectively (Doya, 1999, 2000). Recently, Yamazaki and Lennon (2019) have proposed that the cerebellum is a reinforcement learning machine. These studies suggest that multiple learning systems (supervised and reinforcement learning systems) are driven in parallel in the cerebellum. Large-scale cerebellum and whole-brain simulations would allow us to explore how multiple learning systems work in parallel in the cerebellum and even in the whole-brain. In addition to neurons, experimental evidence on the role of glia cells are accumulating (Ben Haim and Rowitch, 2017). It would be interesting to incorporate neurons and glia cells into the human-scale cerebellum and whole-brain model. We believe that simulations of the models will bring new insights.

## Conclusion

We built a cerebellar network model based on electrophysiological and anatomical data on the K computer using the MONET simulator. The cerebellar network model with the MONET simulator reproduced the activity pattern of granule cells and the OKR simulation as shown by the previous cerebellar network model. Moreover, the MONET simulator showed good weak scaling for our cerebellar network model. Eventually, we demonstrated a human-scale cerebellar network model simulation. These results serve as a fundamental step toward human-scale whole-brain simulations and contribute to our exploration of the computational mechanisms in the human cerebellum.

## Data Availability Statement

The complete JSON file of the computer settings and the neural network parameters for the cerebellar model can be found in the GitHub https://github.com/yamaurahiroshi/MONET.

## Author Contributions

HY, JI, and TY designed the study and, analyzed and interpreted the simulated data; HY performed the simulations; JI developed the neural network simulation software. HY and TY wrote the manuscript.

## Conflict of Interest

The authors declare that the research was conducted in the absence of any commercial or financial relationships that could be construed as a potential conflict of interest.
