# Supplementary material for: Simulation of a Human-Scale Cerebellar Network Model on the K Computer
Source: Front Neuroinform. 2020 Apr 3;14:16. doi: 10.3389/fninf.2020.00016 (PMC7146068; doi:10.3389/fninf.2020.00016)
Supplement: Supplementary file 1 [file Table_1.DOCX]

Supplementary Material for

**Simulation of a human-scale cerebellar network model on the K computer**

Hiroshi Yamaura, Jun Igarashi, Tadashi Yamazaki

1. **Detailed explanation of the similarity index**

Figure S4A shows a schematic raster plot of granule cells (GRs) in response to tonic mossy fiber (MF) signals for 1000 ms, which is modified from our previous paper (Yamazaki and Tanaka, 2007b). GRs repeat to become active (i.e., spiking) and inactive (i.e., nonspiking) alternately. The active periods sustain for tens of milliseconds due to the NMDA receptor-mediated EPSPs, and different GRs show different temporal activity patterns because of the random inhibitory connections with Golgi cells (GOs). GRs in our simulation (Figure S4B left) also show the random alternation of active/inactive periods, although most cells emit spikes tonically and so do not show the alternation. The raster plot is qualitatively different from a random spike pattern artificially generated by Poisson spikes (Figure S4C left), in which such random alternation cannot be seen completely. To confirm this difference, the similarity index is used in the manuscript. Here, however, we define a "similarity matrix" (Yamazaki and Tanaka, 2005), which calculates the similarity (or normalized correlation) between two active populations at *t* = $t_{1}$ and $t_{2}$ ms, as follows:

$SM\left( t_{1}, t_{2} \right)=\frac{\sum_{i} z_{i}\left( t_{1} \right)z_{i}\left( t_{2} \right)}{\sqrt{\sum_{i} z_{i}^{2}\left( t_{1} \right)}\sqrt{\sum_{i} z_{i}^{2}\left( t_{2} \right)}}$ (S1)

Note that the similarity index described in Eq. (2) can be calculated from the similarity matrix as follows:

$SI\left( \Delta t \right)=\frac{1}{T}\sum_{t_{1}=0}^{T} SM\left( t_{1},t_{1}+\Delta t \right)$ (S2)

In the similarity matrix calculated from the actual raster plot, a wide white diagonal band can be seen, which shows the duration of how long an active GR population is kept against the gradual temporal evolution (Figure S4B middle). The similarity index calculates the duration as about 230 ms (Figure S4B right, full-width at the half height). We confirmed that there is no point in the off-diagonal region showing the value of 1, indicating that an active GR population appears only once during the stimulation. In this way, because of the random alternation of active/inactive periods of GRs, the population of active GRs for any given time step is uniquely determined. Furthermore, the same population would never appear more than once due to the recurrent network dynamics by GRs and GOs. These properties cannot be seen in the artificial random spike pattern. The similarity matrix shows only a thin diagonal line and the full-width at the half height is about 64 ms (Figures S4C middle and right, respectively).

**Figure S1.** A representative JSON file for the computer settings

**Figure S2.** A representative JSON file for the neural network parameters. The JSON file has four sections (neural network structure, neuron model parameters, intra-regional connections, and inter-regional connections). The neuron model parameters show an example of the parameters of a granule cell (GR). The session of intra-regional connections shows an example of connection from the GR to Golgi cell (GO).

**Figure S3.** A representative projection area by "orthogornal_cross" function in MONET simulator. Users determine "pre_width" and "post_width" parameters.

**Figure S4.** Details of the similarity index. (**A**) A schematic raster plot of granule cells (GRs) in response to tonic MF signals for 1000 ms (modified from Yamazaki and Tanaka, 2007b). The horizontal axis is time (ms) and vertical axis is neuron number. Only 50 out of 0.1 million GRs are shown. At *t* = 200 ms, GRs shown in red, green, and blue are active, whereas at *t* = 400 ms, these in red, yellow, and purple are active. The red GRs are active at the both time step, whereas green and blue GRs are only at *t* = 200 ms and yellow and purple are only at *t* = 400 ms. Thus, the active GR populations at *t* = 200 and 400 ms are different, although there is some overlap. At *t* = 600 and 800 s, the same GRs shown in red, yellow, and blue are active, but the rest of the active GRs are different (not shown). (**B**) Left: raster plot of the actual spike pattern of GRs (the same with Figure 2A). Conventions as in A. Middle: the similarity matrix calculated from the raster plot. The row and column represent time step $t_{1}$ and $t_{2}$, and the value represents the similarity in Equation (S1). The values are plotted in a gray scale: the lowest in black and the highest (i.e., 1) in white. Right: the similarity index calculated from the similarity matrix. (**C**) Right: raster plot of random spike patterns generated artificially by 50 Hz Poisson spikes. Conventions as in A. Middle and left: the similarity matrix and index for the artificial spike pattern as in B.

**Table S1.** Synaptic weights for optokinetic response

|  |  | Postsynaptic neuron | | |  |  |  |  |  |
| --- | --- | --- | --- | --- | --- | --- | --- | --- | --- |
|  |  | ST | BA | PC | GR | GO | VN | IO | NRTP |
| Presynaptic neuron | ST | 0.02 |  | 0.3 |  |  |  |  |  |
|  | BA |  | 0.02 | 0.4 |  |  |  |  |  |
|  | PC |  | 0.01 |  |  |  | 0.027 |  |  |
|  | GR | 0.0023 | 0.0022 | 0.003 |  | AMPA |  |  |  |
|  |  |  |  |  |  | 0.0006 |  |  |  |
|  |  |  |  |  |  | NMDA |  |  |  |
|  |  |  |  |  |  | 0.00015 |  |  |  |
|  | GO |  |  |  | 3.5 |  |  |  |  |
|  | VN |  |  |  |  |  |  |  |  |
|  | IO |  |  | 0.1 |  |  |  |  |  |
|  | NRTP |  |  |  | 0.6 |  |  |  |  |

ST, stellate cell; BA, basket cell; PC, Purkinje cell; GR, granule cell; GO, Golgi cell; VN, vestibular nucleus; IO, inferior olive cell; NRTP, nucleus reticularis tegmenti pontis.
